# Additive interaction of gefitinib (‘Iressa’, ZD1839) and ionising radiation in human tumour cells *in vitro*

**DOI:** 10.1038/sj.bjc.6602242

**Published:** 2004-11-16

**Authors:** N Giocanti, C Hennequin, D Rouillard, R Defrance, V Favaudon

**Affiliations:** 1 U 612 INSERM & Institut Curie-Recherche, Laboratoires 110-112, Centre Universitaire, 91405 Orsay, France; 2 Cancérologie-Radiothérapie, 1 avenue Claude Vellefeaux, Hôpital Saint-Louis, 75010 Paris, France; 3 Service de Cytométrie, Institut Curie-Recherche, 26 rue d'Ulm, 75005 Paris, France; 4 AstraZeneca-France, 1 place Louis-Renault, 92844 Rueil-Malmaison, France

**Keywords:** EGFR, gefitinib, ionising radiation, cell cycle, cytotoxicity, apoptosis

## Abstract

Cultures of human carcinoma A-431, A-549 and HeLa cells were challenged with *γ*-rays without or with concomitant exposure to gefitinib, a potent inhibitor of the tyrosine kinase activity of epidermal growth factor receptor (EGFR). The outcome of treatment was determined from cell and colony count, cell cycle progression and DNA double-strand break formation and rejoining. Apoptosis was measured in parallel from hypodiploid DNA and using an annexin V assay. Gefitinib developed a cytostatic effect in all cell lines, with drug sensitivity correlating the level of EGFR expression. A weak cytotoxicity of gefitinib was observed in HeLa cells only, although the drug was unable to induce significant cell cycle redistribution in this cell line. In contrast, substantial G1 block and S-phase depletion was observed in A-431 and A-549 cells exposed to gefitinib. The drug brought about additive to subadditive interaction with radiation with regard to growth inhibition, clonogenic death and induction of apoptosis. Consistently, gefitinib did not hinder the rejoining of radiation-induced DNA double-strand breaks in any cell line. The results demonstrate that gefitinib may elicit cytotoxicity at high concentration, but does not act as a radiosensitiser *in vitro* in concomitant association with radiation.

The signalling pathways downstream from the epidermal growth factor receptor (EGFR) play a key role in the control of tumour cell proliferation, angiogenesis and metastatic spread. Actually, activation or overexpression of EGFR has been associated with local recurrence and poor prognosis in patients (Nicholson *et al*, 2001; Gupta *et al*, 2002; Magné *et al*, 2002), as well as with resistance to drugs and radiation in murine carcinomas *in vivo* (Akimoto *et al*, 1999) and human tumour cells *in vitro* (Schmidt-Ullrich *et al*, 1997; Dent *et al*, 1999; Chen *et al*, 2000). Monoclonal antibodies such as cetuximab (IMC-225, Imclone or Erbitux), a human-mouse chimeric antibody directed against the extracellular ligand-binding region of EGFR, and chemical inhibitors of the tyrosine kinase domain of EGFR such as gefitinib (‘Iressa’, ZD1839), a synthetic anilinoquinazoline, have consistently been designed in hopes that they might downregulate mitogenic pathways and provide enhanced response to cytotoxic drugs or ionising radiation.

Preclinical studies of combined treatment with radiation have been performed with both cetuximab and gefitinib. Enhanced radiation susceptibility by cetuximab was reported in a variety of epithelial tumour cell lines *in vitro* (Huang and Harari, 2000; Bianco *et al*, 2002; Culy and Faulds, 2002; Magné *et al*, 2002; Williams *et al*, 2002; Solomon *et al*, 2003). This effect correlated increased radiation-induced apoptosis (Huang *et al*, 1999; Harari and Huang, 2001; Sclabas *et al*, 2003) and S-phase depletion (Huang and Harari, 2000; Harari and Huang, 2001). In combination with radiation, gefitinib brought on synergistic antiproliferative and proapoptotic effects *in vitro* and increased tumour growth delay in the treatment of human tumour xenografts in mouse (Bianco *et al*, 2002; Culy and Faulds, 2002; Huang *et al*, 2002; Williams *et al*, 2002; Solomon *et al*, 2003).

However, the additivity status of gefitinib–radiation interaction *in vitro* has not been firmly established. In fact, the determination of additivity is subject to many drawbacks, which are listed below. (i) Contrary to cytotoxic drugs, the equation fitting the dose dependence of radiation survival includes a quadratic term. Isobolic methods, based on the isoeffect concept, have been designed to overcome this difficulty (Steel and Peckham, 1979), but are not relevant here because the cytotoxicity of EGFR inhibitors is weak. (ii) Contact with cytostatic agents, such as EGFR inhibitors, may result in accumulation of cells in a radiosensitive compartment of the cell cycle (Huang and Harari, 2000). (iii) The presence of cell clusters or multiplets introduces large errors in viability measurements with clonogenic assays, and leads to overestimation of radiosensitivity (Rockwell, 1985) particularly in the low dose range of radiation. This may occur in experiments involving prolonged exposure to cytostatic drugs as growth arrest by these drugs is seldom complete. (iv) To overcome this problem, clusters may be dislocated and replated as isolated cells. However, immediate plating following radiation allows expression of the so-called ‘potentially lethal damage’ and boosts radiation-induced lethality by a large factor (Little *et al*, 1973). (v) A persistent drop of the proliferation rate is frequently observed among radiation survivors.

Taking into account the above pitfalls, we re-evaluated the interaction between gefitinib and radiation in three human tumour cell lines (A-439, A-541, HeLa) differing in EGFR expression, with reproductive cell death, altered cell cycle progression and DNA double-strand break induction and rejoining as end points. The results show that gefitinib may elicit a cytotoxic potential in some cell lines but does not impair radiation recovery.

## MATERIALS AND METHODS

### Products

Gefitinib was kindly provided by AstraZeneca (Rueil-Malmaison, France) as a micronised powder. Aliquots were stored as a 10 mM stock solution in pure dimethyl sulphoxide (DMSO), and dilutions were made daily in growth medium. The final concentration of DMSO (0.2%) was low enough so as not to alter cell growth. Rabbit polyclonal antibody raised against EGFR came from Cell Signaling Technology (Beverly, MA, USA). Protease inhibitors and anti-*α*-tubulin mouse monoclonal antibody were from Sigma-Aldrich Co. (Saint Quentin Fallavier, France). Goat HRP-conjugated secondary antibodies were from Jackson ImmunoResearch Laboratories (West Grove, PA, USA). All products for cell culture were from Invitrogen (Cergy-Pontoise, France).

### Cell lines

A-431 (human vulvar carcinoma, ATCC CRL-1555; p53 mutated), A-549 (human lung carcinoma, ATCC CCL-185; wild type p53) and HeLa cells (human cervix carcinoma, kindly provided by Dr J Coppey; nonfunctional p53 in relation to HPV16-E6 expression) were grown (37°C, 93% air with 7% CO_2_) as monolayers in RPMI-1640 (A-431, A-549) or Dulbecco's minimal essential medium (HeLa) supplemented each with 10% foetal calf serum, 100 IU ml^−1^ penicillin, 0.1 mg ml^−1^ streptomycin and 0.86 mg ml^−1^ Glutamax-I.

A-431 cells reportedly express high levels of EGFR (Ullrich *et al*, 1984; Christensen *et al*, 2001; Moasser *et al*, 2001). Epidermal growth factor receptor expression is comparatively low in A-549 (Sirotnak *et al*, 2000; Moasser *et al*, 2001) and HeLa cells (Hu *et al*, 1997; Bandyopadhyay *et al*, 1998). This was confirmed by Western blot analysis (data not shown) in cells grown under the same conditions (10% foetal calf serum) as used for gefitinib and radiation assays. The relative amounts of EGFR were 15.3 (A-431), 1.0 (A-549) and 1.9 (HeLa).

### Irradiation

*γ*-Ray irradiation of cells with or without concomitant exposure to gefitinib was performed at room temperature (21–24°C) using an IBL-637 (^137^Cs) irradiator (CIS-Biointernational). The dose rate was 1.0 Gy min^−1^.

### Cytotoxicity and growth inhibition

The response of cells to radiation (up to 8 Gy), gefitinib (up to 19 *μ*M), or a combination of both, was determined using colony-forming assays. Dimethyl sulphoxide (0.2%) was present at constant concentration in all experiments. For treatments involving short drug exposure, 800–1000 cells from exponentially growing subcultures were isolated by trypsinisation, plated in triplicate in 25-cm^2^ flasks and incubated at 37°C for 4 h prior to treatment. Following treatment, the flasks were rinsed twice with Hank's balanced salt solution, and cells returned to normal growth medium for 11 or 12 days. Colonies were fixed with methanol, stained and scored visually. The surviving fraction was calculated as the percentage of colonies relative to controls.

As cultures were exposed for 24 h or more to gefitinib, cells had to be plated after treatment. In combined treatment with *γ*-rays, cells were allowed to recover from radiation for 24 h prior to trypsin, counted and plated at a density of 800–1000 cells (25 cm^2^ flasks).

For growth inhibition assays, 25 cm^2^ flasks were seeded with 10^5^ cells and incubated for 24 h prior to introduction of gefitinib. Cells were harvested and counted every day for 8 days after drug, with or without combined irradiation. The drug was renewed every 48 h.

### Cell cycle analysis

Cell cycle progression was monitored by dual-parameter flow cytometry using a FACStar PLUS cytofluorometer (Bekton-Dickinson Biosciences, Le Pont de Claix, France). Cells were grown for 1–6 days with or without gefitinib. At 20 min before harvesting, cells were incubated with BrdUrd (10 *μ*M) for pulse labelling of S-phase cells. Floating and adherent cells were pooled after trypsinisation, and fixed in 70% ice-cold ethanol. Treatment of fixed cells, data acquisition and processing were performed as described (Demarcq *et al*, 1992). Cell cycle analysis was performed with ProCyt software (CEA-INSERM, Grenoble, France).

### DNA double-strand break determination

DNA double-strand breaks were measured by neutral filter elution as described (Bradley and Kohn, 1979; Pommier *et al*, 1984). Cells were labelled with [2-^14^C]thymidine (0.06 *μ*Ci ml^−1^) for 48 h, allowed to ligate Okasaki fragments prior to treatments and sequentially lysed by 0.2% *N*-lauroylsarcosine, 0.2 M NaCl, followed by 2% laurylsulphate and 1 mg ml^−1^ proteinase K for 30 min. The retention of [^14^C]thymidine-labelled DNA was measured after 20-ml (10-h) elution.

### Determination of apoptosis

Apoptosis was determined on floating and adherent cells using two techniques.

*Analysis of DNA fragmentation by flow cytometry*: The hypodiploid (sub-G1) fraction was measured using FACS analysis of propidium-iodide-stained cells after overnight fixation with cold 70% ethanol. The sub-G1 region was determined by a gate on the DNA content histogram excluding the debris at the origin of the abscissa.
*Annexin V assay*: Detection of phosphatidylserine on the outer face of the plasma membrane was carried out on fresh cells with FITC-conjugated Annexin V (Oncogene Research Products, San Diego, CA, USA). 10^6^ cells were suspended in 1 ml ice-cold binding buffer (10 mM HEPES, 150 mM NaCl, 2.5 mM CaCl_2_, 1 mM MgCl_2_, 4% BSA, pH 7.4). A volume of 10 *μ*l of media-binding buffer and 1.25 *μ*l of Annexin V-FITC (200 *μ*g ml^−1^) were added to 500 *μ*l of the cell suspension for 15 min at room temperature, in the dark. Cells were harvested by centrifugation and resuspended in 0.5 ml cold binding buffer. A first analysis by flow cytometry was immediately performed with cells stained with Annexin V-FITC only. Propidium iodide (10 *μ*l; 30 *μ*g ml^−1^ in PBS) was subsequently added and analysis of both PI and FITC fluorescence was carried out with CellQuest Pro software (Becton-Dickinson Biosciences) as described (Darzynkiewicz *et al*, 1997). Cells that bound Annexin V-FITC without propidium iodide staining were considered as early apoptotic cells; necrotic or apoptotic cells in terminal stages were positive for both annexin V-FITC and propidium iodide.

### Western blot analysis

2 × 10^7^ mid-log growing cells were lysed in 250 *μ*l RIPA buffer supplemented with 1 mM phenylmethanesulphonyl fluoride plus protease inhibitors. Proteins were titrated using the Bio-Rad's DC protein assay (Bio-Rad, Hercules, CA, USA). After SDS–polyacrylamide gel electrophoresis, the proteins were blotted onto nitrocellulose membrane (Scleicher & Schuell, Dassel, Germany), incubated with specific primary and secondary antibodies and revealed using an ECL kit. The densitometric analysis of films was carried out with the aid of QuantityOne software (Bio-Rad).

## RESULTS

### Growth inhibition and cell cycle redistribution by gefitinib

Cells were incubated in the presence of increasing concentrations of gefitinib for up to 8 days, and growth measured by cell count relative to untreated samples. Gefitinib alone produced a concentration-dependent inhibition of cell proliferation in the three cell lines. IC_50_, that is, the drug concentration that reduced growth to 50% of that in controls (5-days contact with drug) was 0.3, 6 and 8 *μ*M for A-431, A-549 and HeLa cells, respectively.

**Figure 1:**
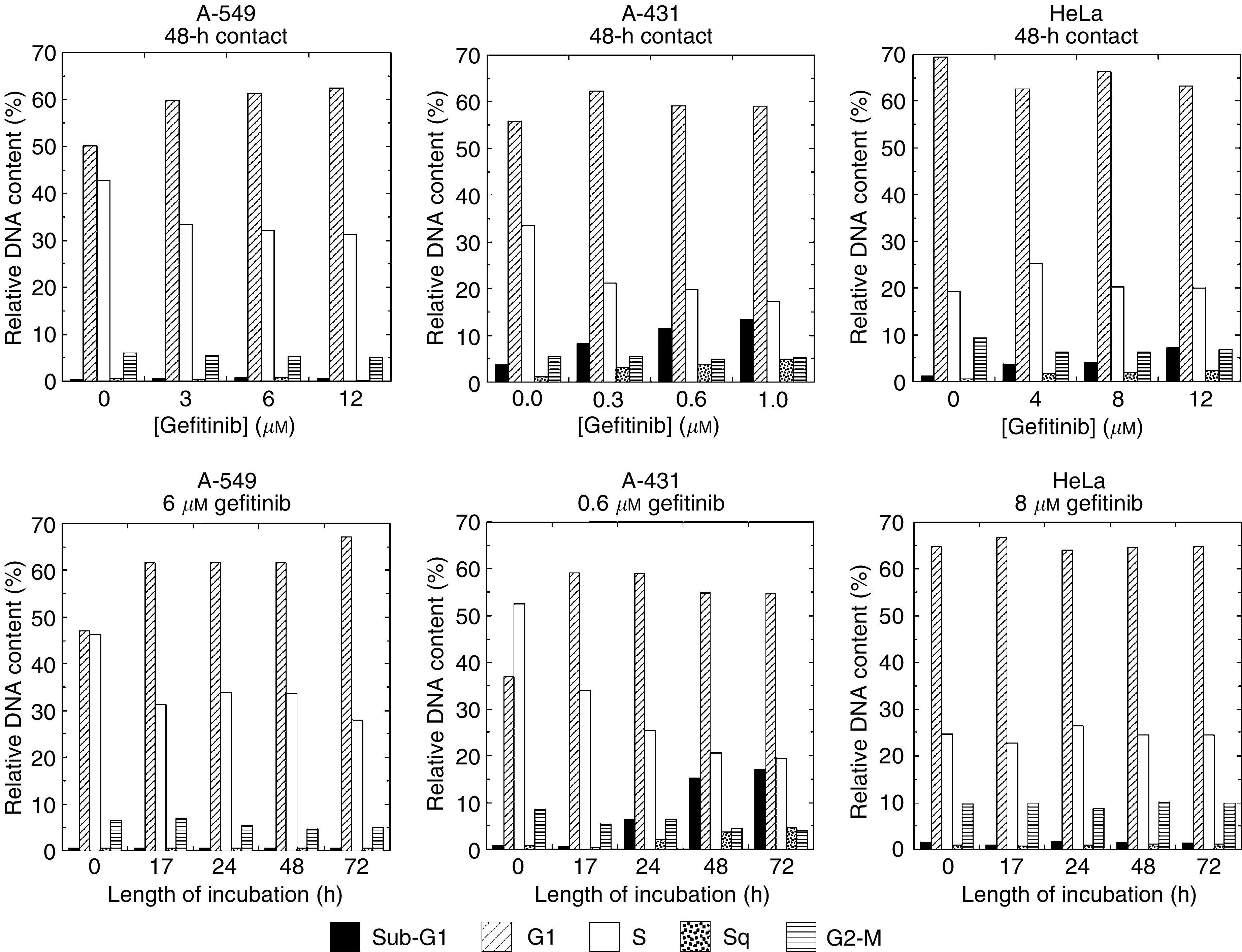
Altered cell cycle progression by gefitinib. A-549, A-431 and HeLa cells were exposed to various concentrations of gefitinib for up to 5 days. Cells were harvested daily after BrdUrd labelling, fixed and analysed by flow cytometry. Sq is the percentage of cells arrested during S phase. The top row shows the concentration-dependent drug response after 48-h incubation. The bottom row shows the evolution of the various compartments of the cell cycle in the presence of an IC_50_ of drug.

Cell cycle disruption by gefitinib was investigated by flow cytometry as a function of both the drug concentration and length of incubation. Gefitinib brought about G1-phase accumulation and S-phase depletion in A-549 and A-431 cells only (Figure 1

). Above the IC_50_, the strength of this effect depended more on the length of contact with the drug than on the drug concentration, and was as anticipated from earlier studies (Bianco *et al*, 2002; Huang *et al*, 2002; Solomon *et al*, 2003). In addition, substantial accumulation of hypodiploid (sub-G1) nuclei was observed specifically in A-431 cells in proportion to the drug concentration and the length of incubation (see below). Minor amounts of hypodiploid nuclei were also observed in HeLa cells, although gefitinib did not perturb cell cycle progression in this cell line.

### Cytotoxicity of gefitinib and phenotype alteration

The cytotoxic potential of gefitinib in short exposure (2-h), was assessed by clonogenic assays in the three cell lines of interest. No cytotoxicity was observed in A-431 and A-549 cells. In contrast, gefitinib developed weak, concentration-dependent cytotoxicity (IC_50_≈51 *μ*M) against HeLa cells.

Prolonged contact with gefitinib at high concentration resulted in marked alterations in cell morphology. Cells flattened with an increased cytoplasm-to-nucleus surface ratio by a factor of 2.0±0.3, and A-549 and A-431 cells at the periphery of colonies extended large lamellipodes. This might explain why, in a few instances, a substantial part of cells was killed by trypsin after prolonged exposure to gefitinib.

### Effect of gefitinib on radiation survival: clonogenic assays

**Figure 2:**
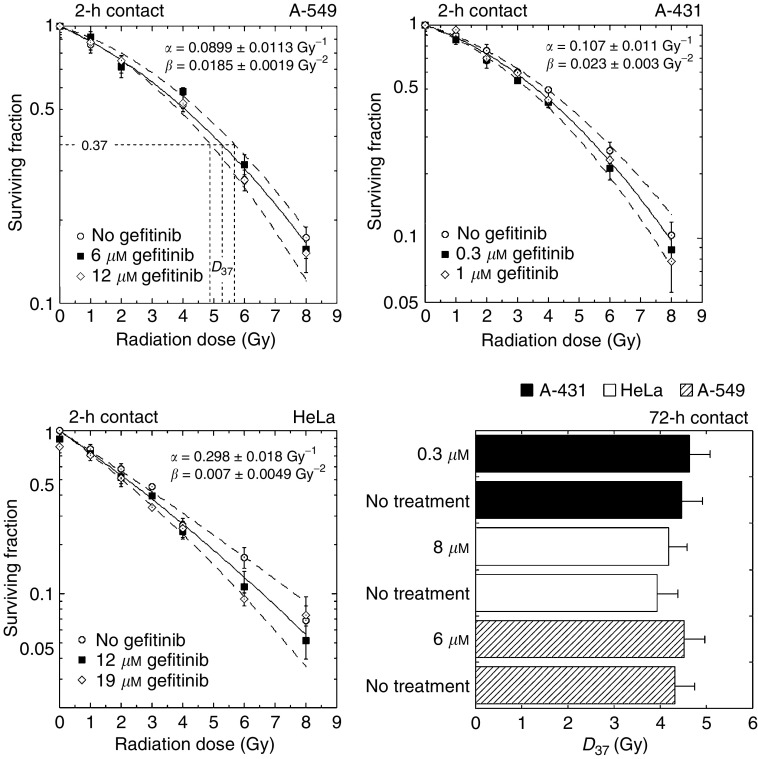
Summary of clonogenic radiation response following short (2-h) and prolonged (72-h) contact with gefitinib. For 2-h contact (survival curves), cells were plated in triplicate before treatment and exposed to gefitinib for 1-h preceding and following irradiation. The surviving fraction (S) was fitted to the classical linear–quadratic equation, ln S=–*αD*–*βD*^2^, where *D* is the radiation dose and *α* and *β* adjustable parameters. Calculations were made through nonlinear least-squares regression taking all data points into account, using Kaleidagraph software (Synergy Software, Reading, PA, USA). Bars represent standard deviation. The dotted lines were drawn according to the linear–quadratic model from the *α*_max_, *β*_max_ and *α*_min_, *β*_min_ couples, respectively, and represent the limit of confidence deduced from standard deviation. For HeLa cells, the minor drop in survival at null radiation dose corresponds to the cytotoxicity of gefitinib against this cell line. For 72-h contact (bar diagram, bottom right), cells were exposed for 48 h to gefitinib, irradiated, incubated with drug for a further 24 h, trypsinised and plated at a suitable density. Survival curves were fitted to the linear–quadratic model. The *D*_37_ (or *D*_0_) values, that is, the doses that reduce survival to 1/*e*=0.37 of that in controls, were calculated from these curves (see upper left panel). The results suggest a weak trend to increased radioresistance by gefitinib.

To determine whether gefitinib was able to alter radiation survival, cells were exposed to gefitinib for 1 h, irradiated and returned to the incubator for a further 1 h in the presence of drug. No modification of radiation response was observed in any of the three cell lines (Figure 2

).

A different protocol was used to study the effect of prolonged contact (>24 h) with gefitinib. In that case, cells were trypsinised and plated at a known density long enough after irradiation (24 h) to allow the repair of potentially lethal damage. No significant difference was observed between radiation alone and combined treatment, though a trend towards a minor increase of radioresistance was noted (Figure 2). Finally, no change in radiosensitivity was observed as the length of drug contact preceding and following irradiation was raised from 1 to 48 h.

### Effect of gefitinib on radiation survival: growth inhibition

Plated cells were allowed to grow for 24 h, then incubated for 48 h with gefitinib, irradiated and harvested after an additional 1–6-days incubation in the presence of gefitinib at the same concentration. The cell count was monitored daily.

Figure 3

**Figure 3:**
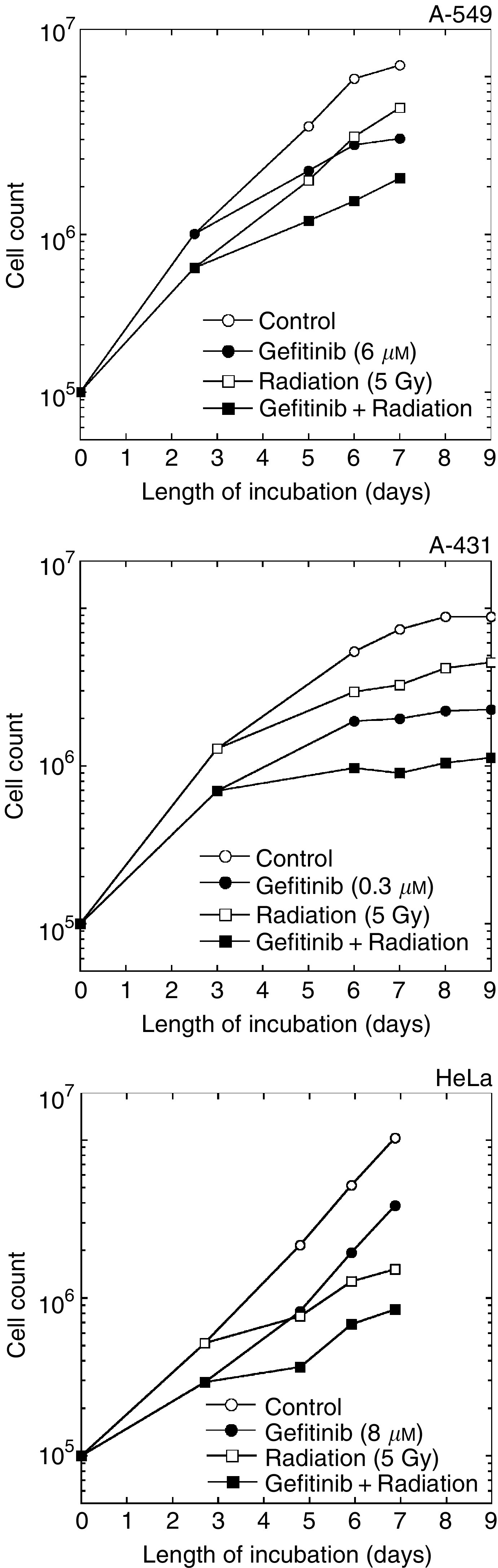
Growth inhibition by gefitinib and radiation. Cells were exposed to gefitinib for 48 h, irradiated and subsequently incubated with gefitinib for up to 7 days. Note that radiation survivors did not recover a full growth potential. Gefitinib induced an additional cytostatic effect, in such a way that growth curves with and without drug were parallel.

shows the results from cells exposed to an IC_50_ of gefitinib. Gefitinib exerted a cytostatic effect, as expected. Persistent attrition of the reproductive potential, in addition to induced kill, was observed in irradiated cells. This, notwithstanding cell proliferation relative to control in combined treatment, matched exactly the product of the growth-inhibitory effects of radiation and gefitinib applied independently, indicating strict additivity (Table 1

**Table 1:** Cell proliferation relative to control for gefitinib, radiation or a combination of both

	**Treatment**	**A-431**	**A-549**	**HeLa**
a	Gefitinib	0.253 (0.3 *μ*M)	0.343 (6 *μ*M)	0.470 (8 *μ*M)
b	Radiation (5 Gy)	0.473	0.538	0.161
c	Product *a* × *b*	0.120	0.184	0.076
d	Combined treatment	0.119	0.192	0.082

**Figure 4:**
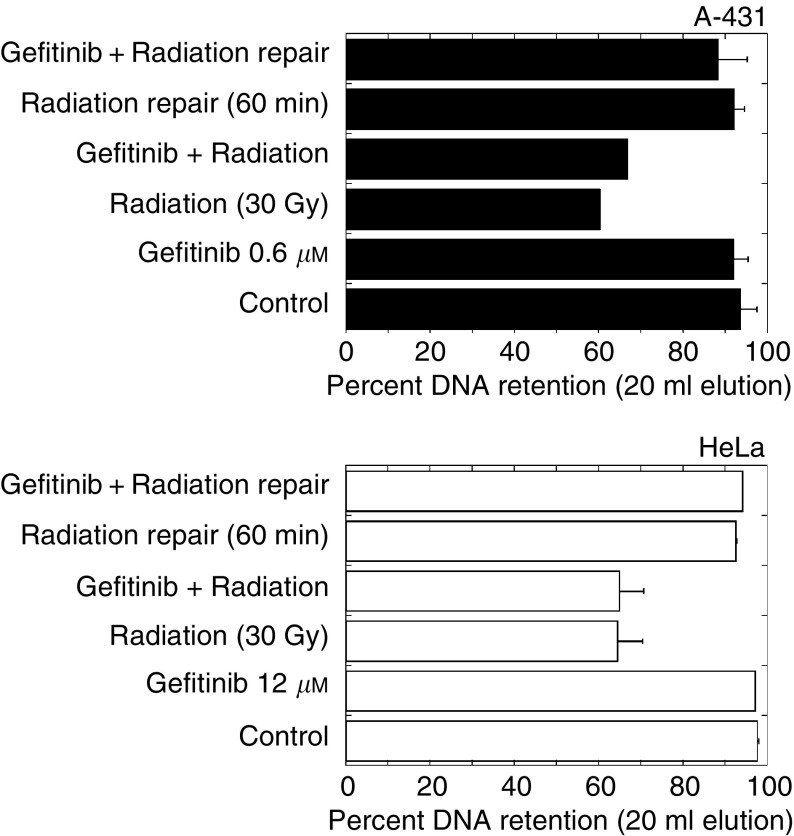
Neutral elution analysis of DNA double-strand break formation and repair after exposure to gefitinib (3-h contact), irradiation (30 Gy) or a combination of both. In combined treatment, cells were exposed to gefitinib for 2 h, irradiated and lysed immediately or after 1-h recovery at 37°C. Each experiment was performed in duplicate. Bars, mean deviation.

Treatments were as in Figure 4, and cells were counted at day 7 after the beginning of treatment.

).

### Effect of gefitinib on DNA double-strand break induction or repair

Epidermal growth factor receptor-dependent transduction has been reported to generate activation, enhance transcription and nuclear translocation of DNA-dependent protein kinase (DNA-PK) in A-431, HeLa and SCC-13Y cells (Bandyopadhyay *et al*, 1998; Huang *et al*, 2002). Cetuximab has also been reported to trigger binding of EGFR to the heterotrimeric DNA–PK complex in the cytoplasmic fraction and deplete the nuclear pool of DNA–PK (Bandyopadhyay *et al*, 1998). Whether this elicits impaired DSB repair and increased radiation susceptibility *in vivo* is open to question, as phase I studies did not show limiting toxicity of cetuximab in association with radiotherapy against advanced head and neck cancer (Robert *et al*, 2001). It may be best to consider that nuclear exclusion of DNA-PK results from S-phase depletion by the antibody. Actually, cetuximab causes a marked G1 block (Huang and Harari, 2000), while nuclear import and activation of DNA-PKcs occur only in S phase and at the G1/S junction (Lee *et al*, 1997; Nilsson *et al*, 1999). Gefitinib might induce the same effect.) As DNA-PK plays a key role in DNA double-strand break repair through NHEJ, the effect of gefitinib on the rejoining of radiation-induced breaks was investigated by neutral filter elution. Gefitinib alone did not induce any DNA damage. When tested in A-431 and HeLa cells, gefitinib had no effect on the incidence or rejoining of DNA double-strand breaks (Figure 4

).

### Apoptosis in combined treatment with radiation and gefitinib

Apoptosis by gefitinib without and with combined exposure to radiation was investigated in the three cell lines. No apoptosis was detected in A-549 cells, confirming that A-549 cells are resistant to this mode of cell death (Lawrence *et al*, 2001; Stuschke *et al*, 2002). Therefore, the results presented below are for A-431 and HeLa cells only. Two methods were used to assess apoptosis. In each instance, cells were pre-incubated for 48 h with an IC_50_ of gefitinib, prior to *γ*-ray irradiation. Irradiated cells were collected after a further 24-, 48- or 72-h incubation, long after completion of DNA repair.

**Figure 5:**
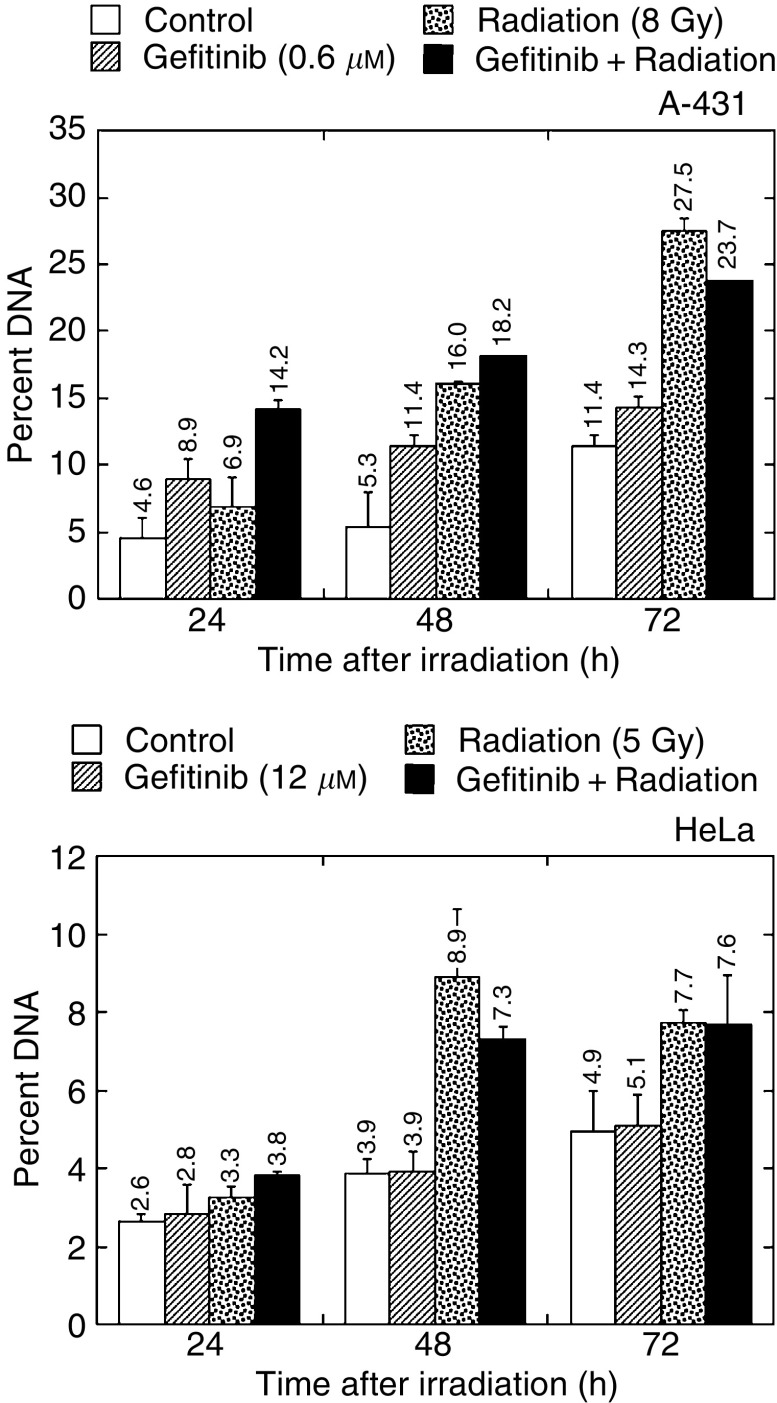
Flow cytometric analysis of the hypodiploid (sub-G1) DNA content in A-431 and HeLa cells exposed to gefitinib, radiation or a combination of both. Cells were exposed to gefitinib for 60 h, irradiated, returned to the incubator in the presence of gefitinib and collected at the times indicated. Mock-irradiated samples were collected after the same length of incubation.

**Figure 6:**
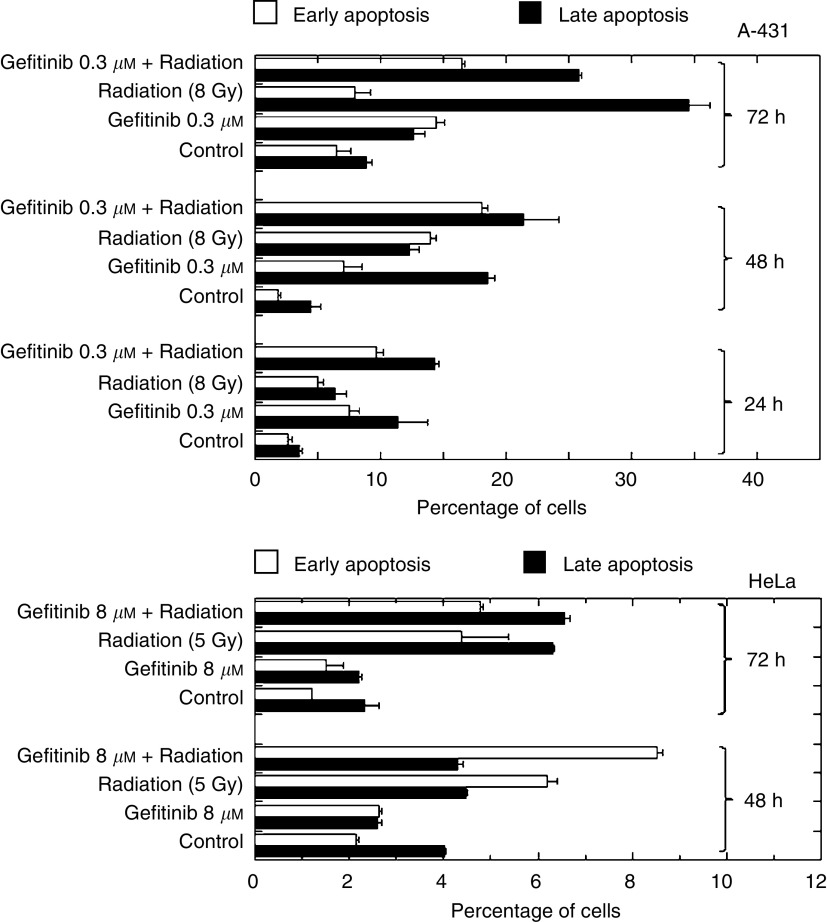
Annexin V-FITC analysis of apoptosis in A-431 and HeLa cells. Cells were exposed to gefitinib for 60 h, irradiated, returned to the incubator in the presence of gefitinib and collected at the times indicated. Nonirradiated, drug-free controls were collected after the same length of incubation. The distinction between early and late apoptosis was made as described (Darzynkiewicz *et al*, 1997).

Gefitinib generated hypodiploid (sub-G1) nuclei in A-431 and HeLa cells, though to a much lower extent in the latter. Ionising radiation also induced an increase of the sub-G1 fraction. In both cell lines, combined treatment produced additive interaction at 24 h. In contrast, infra-additive interaction was observed at 48 and 72 h of post-irradiation incubation in both cell lines (Figure 5

).
FACS analysis of annexin V-FITC binding to the cells' plasma membrane correlated with the sub-G1 content (Figure 6

). Gefitinib alone induced significant apoptosis above background in A-431 cells only. Radiation-induced apoptosis was observed in both A-431 and HeLa cells, but combined treatment did not promote apoptosis. In A-431 cells, nearly additive interaction was observed at 24 h, but infra-additive interaction occurred at 48 and 72 h of post-irradiation incubation in both cell lines.

## DISCUSSION

We found that short (2-h) exposure to gefitinib had no effect on radiation response in clonogenic assays, and strictly additive interaction was observed (Figures 2 and 3) after 72-h contact with gefitinib. Consistently, gefitinib did not impair DNA double-strand break rejoining (Figure 4) and did not interfere with the radiation-induced G2-M block (data not shown). In our hands, gefitinib and radiation acted additively to induce apoptosis in A-431 and HeLa cells after 24-h contact with drug, whereas the interaction was infra-additive (possibly antagonistic) after more prolonged exposure (Figures 5 and 6). However, significant amounts of gefitinib-induced apoptosis were observed in A-431 cells only. In spite of the fact that HeLa cells elicited cytotoxic response to gefitinib, drug-induced apoptosis in HeLa cells was below 5%; A-549 cells did not show evidence of apoptosis. Accordingly, the apoptotic potential and the growth-inhibitory efficiency of gefitinib are not inter-related in the three cell lines used.

HeLa cells did not show significant cell cycle redistribution by gefitinib. In contrast, and in agreement with others (Bianco *et al*, 2002; Huang *et al*, 2002; Williams *et al*, 2002), we observed that in A-431 and A-549 cells gefitinib induced accumulation in G1 at the expense of S phase. Moreover, radiation potentiated growth arrest by the drug (Figures 1 and 4). S phase has long been known to be the most radioresistant compartment of the cell cycle (Terasima and Tolmach, 1963). Therefore, as pointed out by Huang and Harari (2000), S-phase depletion by gefitinib could eventually lead to increased radiosensitivity in cells responding to gefitinib. Indirect evidence in favor of this scheme was provided by Wollman *et al* (1994), who showed that stimulation of MCF-7 cells by extraneously added EGF produced a substantial increase in both S-phase content and radioresistance. However, despite significant S-phase depletion in A-431 and A-549 cells (Figure 1), no radiosensitisation was observed after 72-h contact with gefitinib (Figure 3).

In conclusion, gefitinib at cytostatic concentration did not impair the rejoining of radiation-induced DNA double-strand breaks, and brought about additive interaction with radiation in terms of growth inhibition or induced cell death in A-431, A-549 and HeLa cells. Such additivity may be beneficial in chemo-radiotherapeutic combination. Indeed, supra-additive interaction may lead to acute hypertoxicity, reduction of the maximum tolerated doses of both drug and radiation and treatment failure. Contrary to a widely held opinion, radiosensitisation should be considered with care when it comes to eliciting inhibition of radiation recovery (Balosso *et al*, 1995). Well-known examples of limiting toxicities are from adriamycin (Mayer *et al*, 1976) and bleomycin (Peters *et al*, 1988).

It should be recalled here that the most useful mechanisms in chemo-radiotherapeutic combinations are spatial cooperation, reoxygenation and inhibition of tumour repopulation. Reduction in tumour volume after chemotherapy, when it occurs, may result in improved blood supply to the tumour, leading to reoxygenation and increased radiosensitivity. Furthermore, inhibition of neoangiogenesis may lead to increased radiocurability (Brown, 2001; Hennequin and Favaudon, 2002), and in fact recent studies have convincingly shown that both cetuximab and gefitinib are able to afford potentiation of radiotherapy and inhibition of neoangiogenesis in human tumour xenografts in mice (Bianco *et al*, 2000, 2002; Huang and Harari, 2000; Milas *et al*, 2000; Buchsbaum *et al*, 2002; Huang *et al*, 2002; Williams *et al*, 2002). This might allow gefitinib and radiotherapy to be used concomitantly or in close temporal proximity to take advantage of inhibition of tumour neoangiogenesis or altered cell cycle distribution, without the acute or late healthy tissue complications that are at risk with inhibition of DNA repair.
